# Psychoacoustic Assessment to Improve Tinnitus Diagnosis

**DOI:** 10.1371/journal.pone.0082995

**Published:** 2013-12-12

**Authors:** Charles-Édouard Basile, Philippe Fournier, Sean Hutchins, Sylvie Hébert

**Affiliations:** 1 School of Speech Pathology and Audiology, Université de Montréal, Montréal, Québec, Canada; 2 International Laboratory for Research on Brain, Music, and Sound (BRAMS), Université de Montréal, Montréal, Québec, Canada; 3 Centre de recherche de l’Institut Universitaire de Gériatrie de Montréal (CRIUGM), Montréal, Québec, Canada; University of Salamanca- Institute for Neuroscience of Castille and Leon and Medical School, Spain

## Abstract

The diagnosis of tinnitus relies on self-report. Psychoacoustic measurements of tinnitus pitch and loudness are essential for assessing claims and discriminating true from false ones. For this reason, the quantification of tinnitus remains a challenging research goal. We aimed to: (1) assess the precision of a new tinnitus likeness rating procedure with a continuous-pitch presentation method, controlling for music training, and (2) test whether tinnitus psychoacoustic measurements have the sensitivity and specificity required to detect people faking tinnitus. Musicians and non-musicians with tinnitus, as well as simulated malingerers without tinnitus, were tested. Most were retested several weeks later. Tinnitus pitch matching was first assessed using the likeness rating method: pure tones from 0.25 to 16 kHz were presented randomly to participants, who had to rate the likeness of each tone to their tinnitus, and to adjust its level from 0 to 100 dB SPL. Tinnitus pitch matching was then assessed with a continuous-pitch method: participants had to match the pitch of their tinnitus to an external tone by moving their finger across a touch-sensitive strip, which generated a continuous pure tone from 0.5 to 20 kHz in 1-Hz steps. The predominant tinnitus pitch was consistent across both methods for both musicians and non-musicians, although musicians displayed better external tone pitch matching abilities. Simulated malingerers rated loudness much higher than did the other groups with a high degree of specificity (94.4%) and were unreliable in loudness (not pitch) matching from one session to the other. Retest data showed similar pitch matching responses for both methods for all participants. In conclusion, tinnitus pitch and loudness reliably correspond to the tinnitus percept, and psychoacoustic loudness matches are sensitive and specific to the presence of tinnitus.

## Introduction

The diagnosis of tinnitus relies exclusively on patient self-report and various subjective questionnaires [1-3], thus precluding objective assessment of the progression of the tinnitus percept (with time or therapeutic intervention) and identification of physiological tinnitus at an acceptable level of specificity. As a consequence, much effort has been devoted to devising psychoacoustic measures based on pitch and loudness matching. 

### Pitch matching

Most conventional studies on tinnitus pitch matching designed to identify a single predominant frequency (often described as tonal tinnitus), using either a forced-choice paradigm or an adjustment method [4-24], show that the perceived predominant pitch falls within the frequency band of the hearing loss [10,14,19,20,23]. Because these methods have shown variable degrees of test-retest reliability [4,5,12,15], pitch matching is generally not deemed a good parameter for treatment outcome [25]. Recent studies have used a patient-directed approach with a likeness rating scale [26-35] in which the participants rate the likeness of every frequency (0.25 to 16 kHz by half-octave steps) to their tinnitus, thereby defining a tinnitus spectrum. The likeness rating method showed that tinnitus is composed of a wide frequency bandwidth mirroring the hearing loss region [26,28-31,33,35] even when no hearing loss is found at standard audiometric frequencies (0.25 to 8 kHz) [27,34]. However, it remains unclear whether the likeness rating technique, which involves a discrete mode of presentation, can provide an accurate estimate of the predominant pitch compared to when only one pitch is matched, such as in the continuous-pitch paradigm proposed herein. A first goal of this study was to introduce a new patient-directed tinnitus likeness rating procedure and compare its precision with a continuous-pitch presentation method, while controlling for participants’ musical expertise. Moreover, we conducted test-retest trials to establish the method’s reproducibility.

### Loudness matching

When tinnitus loudness is estimated by adjusting the volume of a single external pure tone to the loudness of the predominant tinnitus pitch, it usually ranges from 5 to 15 dB Sensation Level, or dB SL, even though patients subjectively describe their tinnitus as being very loud [19,36,37]. Some studies have shown good loudness test-retest reliability over a period ranging from several days [4,6,15,31,38,39] to several months [12,34], with less than 5 dB difference between sessions, whereas other studies have reported greater variability [16,21,40], putting into question the validity of this measure for tinnitus diagnosis and follow-up. Herein we investigate the proposition that the assessment of frequency likeness ratings over the entire frequency span will increase the reliability of loudness judgments by providing participants with several opportunities to judge tinnitus loudness.

### Differentiating true from false tinnitus

There is currently no measure that discriminates true from false claims of tinnitus at an adequate level of specificity. Since the economic burden of tinnitus to society is substantial [41], it is surprising that very few studies have attempted to address whether psychoacoustic measures such as pitch and loudness are effective criteria for detecting tinnitus simulation [39,40,42,43]. Regarding pitch matching, studies reported lower tinnitus pitch matches for simulated malingerers – that is, participants instructed to pretend that they had tinnitus [39,40,43]. Regarding loudness matching, studies reported *lower* dB SL matches [40], *higher* dB SPL but *no different* dB SL [39], or *higher* dB SL matches for simulated malingerers [42]. Low-frequency loudness matches were found to be the most predictive value for the presence or absence of tinnitus [43]. A final goal was therefore to examine whether pitch and loudness tinnitus matching can detect people without tinnitus.

Summarizing our method and objectives, we used a new participant-directed likeness rating method to match tinnitus pitch and loudness over a wide frequency spectrum (from 0.25 to 16 kHz). We tested two groups of tinnitus participants with different levels of musical training (musicians and non-musicians), as well as a group of simulated malingerers who feigned tinnitus, and we compared external pitch matching ability performances across groups. Predominant tinnitus pitch obtained with the likeness ratings was compared to a method using a single continuous pitch. Pitch and loudness ratings at the predominant tinnitus pitch were used as predictors to address participants’ sensitivity and specificity for presence or absence of tinnitus. Finally, test-retest reliability was assessed after a delay of several months to test for reproducibility of findings, stability of the tinnitus percept, and suitability for treatment outcome. 

## Methods

### Participants

A total of 50 participants were recruited through newspaper and online ads, and word of mouth. They were either musicians (n=16) or non-musicians (n=16) with tinnitus, or simulated malingerers (n=18) —that is, individuals without tinnitus instructed to simulate this sound perception with the intention of convincing the experimenter that they have tinnitus. Simulated malingerers had to have had previous experience of transient tinnitus, lasting no longer than one day and not in the month prior to the testing, so that they could rely on this past experience to fake tinnitus. Musicians were selected on the criterion of having at least three years of formal musical training (mean = 10 years ± 5.5); otherwise, they were considered non-musicians (mean = 0.13 year ± 0.5). Tinnitus in both groups had to be continuously present for at least six months (mean for musicians = 10.6 ± 7 years; mean for non-musicians = 11.3 ± 11 years). Exclusion criteria were having more than a moderate hearing loss at any standard audiometric frequency in either ear (>55 dB HL for 0.25 to 8 kHz), uncontrolled medical conditions, outer and middle ear pathology, and heavy smoking (> 10 cigarettes/day). The participants’ relevant sociodemographic characteristics are summarized in Table 1A. Overall, at standard frequencies (0.25 to 8 kHz) non-musicians had higher hearing thresholds in the right ear than did simulated malingerers but not than musicians. Non-musicians had also higher thresholds did than musicians and simulated malingerers in the left ear, but the last two did not differ. At very high frequencies (9 to 16 kHz), non-musicians had higher thresholds than did both musicians and simulated malingerers, and the last two also differed from one another. 

**Table 1:** Demographic characteristics (standard deviation) of musicians with tinnitus, non-musicians with tinnitus, and simulated malingerers, at test (A) and retest (B).

**A**	**Musicians**	**Non-musicians**	**Simulated malingerers**	***p*-Value**
N	16	16	18	
Sex (male/female)	13/3	9/7	7/11	.014
Age in years	33 (9.9)	43 (8.5)	23 (2.0)	<.001
Education level in years	19 (3.2)	17 (2.2)	17 (1.9)	n.s.
Tinnitus type (tonal/noise)	11/5	13/3	14/4	n.s.
Tinnitus ear (left/right/central)	3/1/12	1/1/14	1/4/13	n.s.
THQ handicap total score in %	17.4 (12.6)	34.5 (17.2)	43.3 (21.4)	<.001
PTA Standard left ear	8.9 (1.7)	16.3 (1.7)	4.2 (1.6)	<.001
PTA Standard right ear	9.1 (1.6)	14.5 (1.6)	3.9 (1.5)	<.001
PTA VH left ear	19.7 (3.8)	34.0 (3.8)	3.1 (3.6)	<.001
PTA VH right ear	20.3 (3.8)	35.9 (3.8)	5.0 (3.6)	<.001
**B**	**Musicians**	**Non-musicians**	**Simulated malingerers**	***p*-Value**
N	9	9	10	
Sex (male/female)	8/1	5/4	4/6	.033
Age in years	37 (10.5)	41 (8.2)	23 (2.0)	<.001
Education level in years	20 (3.8)	17 (1.6)	18 (2.4)	n.s.
Tinnitus type (tonal/noise)	5/4	7/2	8/2	n.s.
Tinnitus ear (left/right/central)	1/1/7	1/0/8	0/2/8	n.s.
THQ handicap total score in %	14.4 (8.6)	33.4 (17.3)	39.9 (23.9)	.012
PTA Standard left ear	9.4 (2.1)	11.3 (2.1)	5.8 (2.0)	.02
PTA Standard right ear	11.8 (2.2)	12.2 (2.2)	5.7 (2.1)	n.s.
PTA VH left ear	26.0 (5.0)	25.0 (5.0)	4.2 (4.7)	.002
PTA VH right ear	30.3 (5.3)	32.0 (5.3)	5.0 (5.0)	<.001

Pure-tone average for standard frequencies (PTA Standard, from 0.25 kHz to 8 kHz) and for very-high frequencies (PTA VH, from 9 kHz to 16 kHz) are in dB HL.

More than half of the participants in each group – nine musicians, nine non-musicians, and ten simulated malingerers – were retested some weeks later (mean of 25 weeks ± 13). All tinnitus participants confirmed that their tinnitus was essentially unchanged across sessions. Relevant sociodemographic characteristics of the retest participants are summarized in Table 1B. Overall, hearing thresholds were significantly higher for both musicians and non-musicians than for simulated malingerers.

 The study was approved by the Ethics Committee of the Université de Montréal, and all participants gave their written informed consent. 

### Materials and Procedure

#### Hearing test

Hearing detection thresholds were assessed in each ear monaurally from 0.25 to 8 kHz in half-octave steps by a clinical audiologist using the standard modified Hughson-Westlake up-down procedure [44] with an AC-40 clinical audiometer (Interacoustics, Assens, Denmark) and ER-3A insert earphones (Aearo Company Auditory Systems, Indianapolis, IN, USA). In addition, very-high frequency thresholds (9 to 16 kHz) were also assessed monaurally in each ear using Sennheiser HDA-200 supra-aural headphones (Sennheiser Electronic GmbH & Co., Wedemark, Germany). The audiometric equipment was calibrated in a soundproof booth using the ANSI S3.6-2004 standard norms. An otoscopic examination was performed before each hearing test to rule out earwax compaction or middle-ear infection. 

#### Tinnitus matching with the likeness rating method

**Figure 1:**
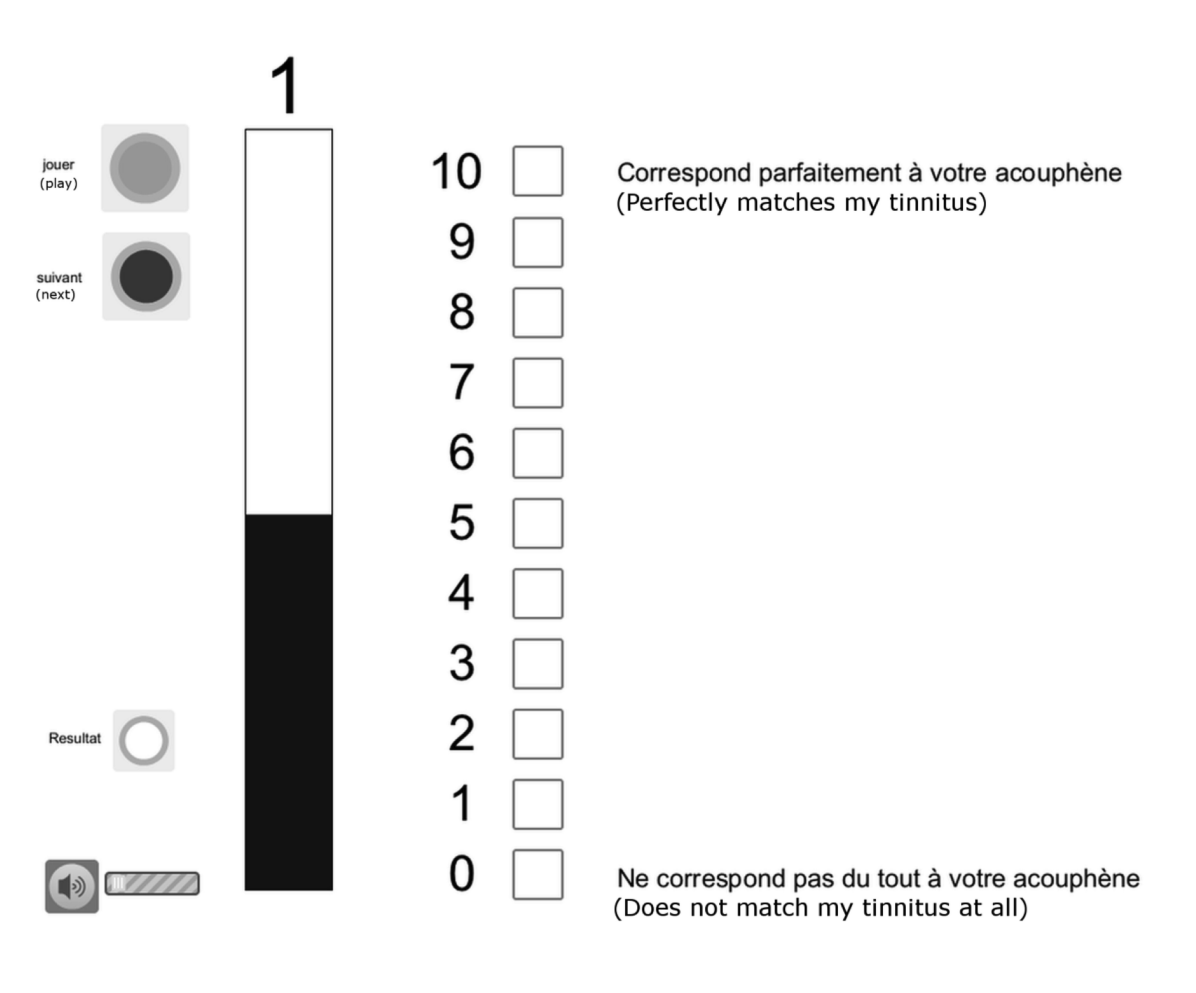
Instructions displayed on the touchscreen for performing tinnitus matching using the likeness rating method. Participants initiated a trial by pressing the green button. They had to rate how the tone contributed to their tinnitus on the 10-point scale. Then, they had to match its loudness by moving the gauge on the left side. When this was done, they could press the red button to initiate the next trial.

The likeness rating method (described in [34]) is a custom-made program running under Max/MSP software (Cycling 74, San Francisco, CA, USA) controlling a visual interface implemented in a computer touchscreen (Élo TouchSystems, Menlo Park, CA, USA). Stimuli were one-second pure tones ranging from 0.25 to 16 kHz (the same frequencies as in the hearing test) generated by a Fireface sound card (RME, Haimhausen, Germany) and presented binaurally using closed DT 770 Pro/250 dynamic headphones (Beyerdynamic, Heilbronn, Germany). Participants sat in a soundproof booth in front of the touchscreen and initiated the presentation of a pure tone by pressing a green button (“Play”) on the screen (Figure 1). They first rated the likeness of the tone to their tinnitus pitch on a Likert-type scale in which 0 = “does not match my tinnitus at all” and 10 = “perfectly matches my tinnitus.” During the same trial, they matched the loudness of the tone—that is, the sound level at which that specific frequency contributed to their tinnitus—by moving a visual gauge that increased and decreased the sound level in 1 dB steps, from 0 to 100 dB SPL. The program allowed participants to play each pure tone as many times as needed. When pitch and loudness matches were completed, participants pressed a red button (“Next”) to initiate the following trial. Each specific pure tone was presented three times in a pseudo-random order such that no two identical frequencies were presented in a row. Two pure tones of 600 Hz and 5 kHz were presented before and served as practice trials. Headphones were calibrated before each session with a SoundPro SE/DL sound level meter using a QE-4170 microphone model (Quest Technologies, Oconomowoc, WI, USA) and an EC-9A 2cc ear coupler (Quest Electronics, Oconomowoc, WI, USA).

#### Tinnitus matching with the slider method

The slider method, described in a previous study [45], was used to validate the precision of the likeness rating method and involved tinnitus pitch matching with a continuous-pitch presentation. Responses were made on a simple device called a slider: two superimposed touch-sensitive strips with the ability to sense pressure and position (Infusion System, Montreal, Canada). These 50-cm strips were mounted on a hard surface, inset between two plastic bars. The slider sent a 10-bit MIDI signal to indicate the position of the participant’s finger press, which was converted into a sine wave by Max/MSP. The frequency of the slider’s output was determined by the position of the participant’s finger press, such that lower tones were created by pressing on the slider’s left side, and higher tones by pressing on the slider’s right side. The range of the slider was fixed for each individual trial, but could be changed between trials. The frequency associated with each position on the slider was based on an exponential curve, such that octaves and semitones were always equidistant in both directions (similar to how tones are arranged on a piano). Although the slider’s output is fundamentally quantized by the position, in practice the 1,024 available positions are so close together (~0.5 mm per position) that the resulting frequency output is perceived as changing continuously when the finger moves between positions. Participants heard the tones generated by a Fireface sound card through DT 770 Pro/250 headphones, and the sound level was adjusted to a comfortable level for each participant by the experimenter.

**Figure 2:**
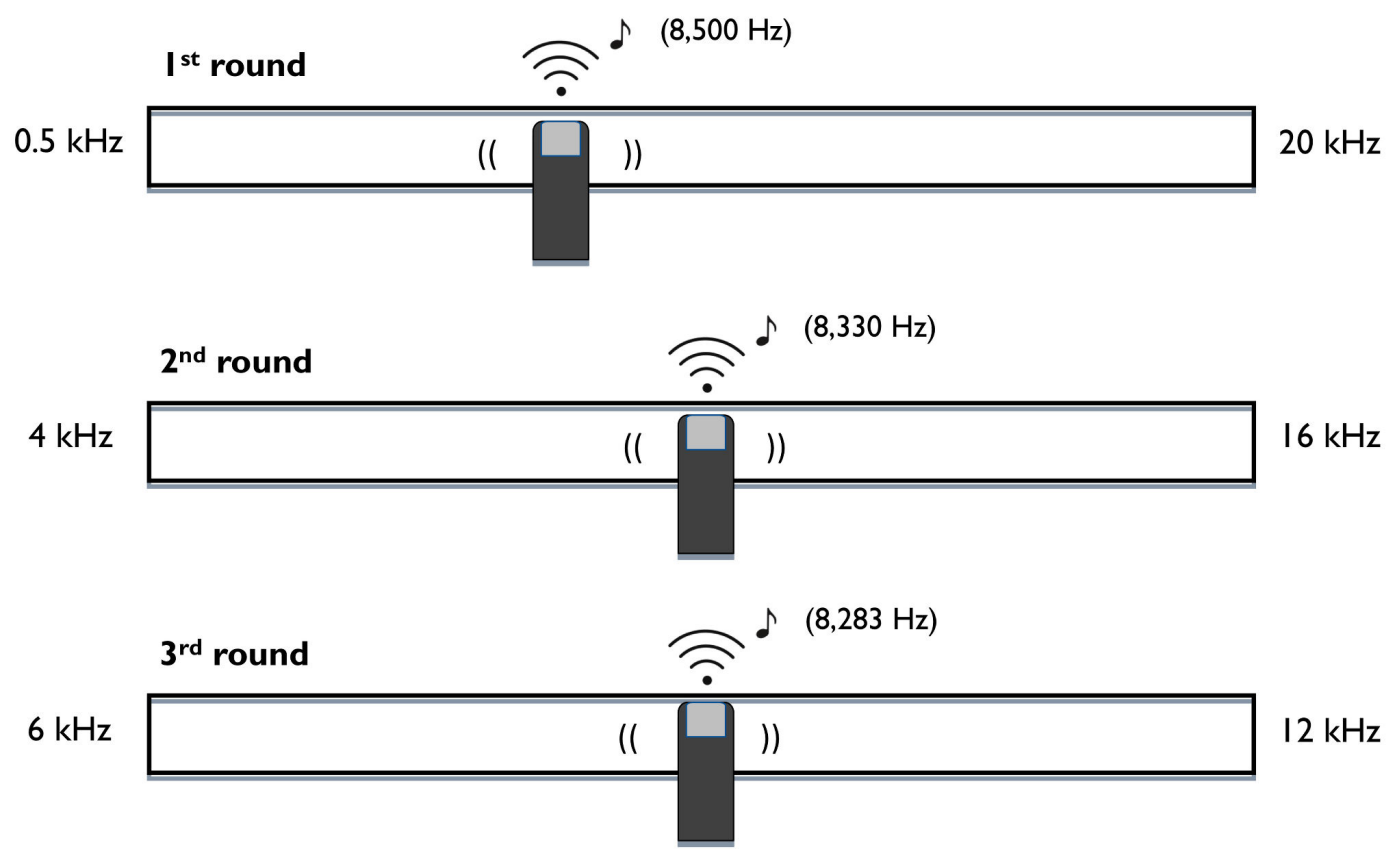
A schematic view of the tinnitus matching procedure using the slider. Each trial included three rounds. In the first round, the slider was set between 500 Hz and 20 kHz for all participants. In the second round, the range was limited to two octaves around the final tone chosen by the participant in round one (here, two octaves around 8 kHz). Once the final tone was chosen in round two, the third round was further limited to one octave around this tone (here, one octave around 8 kHz).

Participants initiated the tinnitus matching trials by pressing the space bar on a keyboard, and they were asked to use the slider to match the pitch of their tinnitus. There were three trials of tinnitus matching, with each trial subdivided into three different rounds (see Figure 2). In the first round, the range of the slider was set between 500 Hz and 20 kHz, to capture the entire possible range of a participant’s tinnitus. Participants were instructed to find the pitch on the slider that best matched their tinnitus, and to save their final response and initiate the following round by pressing the space bar. In the second round, the range was limited to two octaves around the final tone chosen in round one, which was centered on the slider, and participants were again instructed to find the best match for their tinnitus. Once this tone was chosen, the range in the third round was limited to one octave around the final tone chosen in round two, and the tone was once again centered on the slider. This procedure was intended to allow participants to match their tinnitus pitch as specifically as possible, up to 1 Hz precision, while still giving the entire range to draw from. 

#### Pitch matching ability assessment

Using the slider, the ability to match pitches to external tones was assessed. Target tones were set at 0.5 kHz, 2 kHz, 6 kHz, and 14 kHz, to cover most of the range of typical hearing, and were played as sine waves as well. The task began when the participant pressed the space bar on a keyboard. The target tone was played continuously through DT 770 Pro/250 headphones, and participants were instructed to match it as closely as possible on the slider. The target tone was turned off while the slider was being pressed, to avoid using beating or acoustic dissonance as cues, but it was turned back on when the participants removed their finger from the slider. This was done so that the participants never needed to remember the pitch and matching was not impaired by poor pitch memory skills or interference from tinnitus pitch. Participants were told that they could take as long as they liked to match the target. When they had done so, participants saved their final response by pressing the space bar and initiated the next trial. The pitch matching ability task included presentation of 20 tones, using five examples of each target such that no two identical tones were presented in a row. The slider’s total range was one octave during each trial, with the upper and lower boundaries randomly chosen such that the target tone would fall in the middle two thirds of the slider. Thus, participants did not have any cues from prior trials where the target tone would be located on the slider. Furthermore, pure tones of 0.25 kHz, 1 kHz, 4 kHz, 8 kHz, and 12 kHz were previously used as practice trials for the matching task before the target tones were presented. Finally, the loudness of the tone was adjusted to a comfortable level for each participant by the experimenter. 

#### Visual analog scales

Five visual analog scales (VAS) of tinnitus annoyance (“usually,” “now”), loudness (“usually,” “now”), and attention usually spent on tinnitus were used. The scales were 100 mm horizontal lines with the left and right extremes labeled, respectively, “no annoyance at all” and “very annoying,” for all five scales. 

#### Procedure

After hearing threshold assessment, tinnitus matching methods were conducted in a counterbalanced order among groups and between test sessions (test vs. retest). The slider method always began with the pitch matching ability assessment to familiarize the participants with the slider. Participants were asked to provide repeatable tinnitus matching responses to the best of their ability. Simulated malingerers were not instructed to use any particular method to provide consistent responses during matching. All measurements were taken in a soundproof booth at the BRAMS (Eckel Industries, Morrisburg, Ontario, Canada). The validated French version of the Tinnitus Handicap Questionnaire (THQ) [46,47] and the visual analog scales were given in a random order before psychoacoustics tasks. The likeness rating method took 20–30 minutes and the slider method took no longer than 15 minutes.

### Data processing and Statistical Analysis

For the likeness rating method, the mode for each frequency for each participant was used (or the median when the mode failed to reveal a single rating value). Tinnitus loudness matching (in dB SPL) at each frequency was averaged. When participants in a trial rated the likeness of the pure tone as 0, the loudness value of that trial was removed from further analysis. The loudness was converted into dB SL—that is, the difference between the sound pressure level of tinnitus loudness matching and the sound pressure level of the best hearing threshold shift between left and right ears. The predominant frequency of the tinnitus spectrum was defined as the frequency with the highest likeness rating score, and the tinnitus loudness was set as the sensation level value at this frequency. If more than one highest rating value was reported, the predominant tinnitus pitch was averaged between the frequencies corresponding to the lowest and highest rating values across the frequency span and the mean loudness value of those frequencies. 

For the slider method, the predominant tinnitus pitch corresponded to the mean frequency matched in round three of each trial. The ability to match pitch to external tones was assessed by the differences in cents between target tone and matched frequency. Due to his elevated hearing thresholds at 14 kHz, one participant could not match this target tone and was excluded from pitch matching analyses (n=15) at this frequency. Pitch information obtained for the external pitch matching task with the slider was converted to semitones so that meaningful comparisons could be made between different trials. Because responses in both pitch and tinnitus matching tended to consist of multiple instances of discrete tones, the pitch of the final discrete tone produced during each trial was taken as the primary measurement. In the external pitch matching task, the absolute value of the error of the final response (the pitch of final response minus the pitch of target tone) was used to avoid sharp and flat errors canceling out. Final responses for each target tone were considered accurate if the pitch was within 50 cents (1/2 semitone) of the target, a criterion validated in other experiments [48,49]. 

Hearing thresholds were averaged separately for standard frequencies (0.25 to 8 kHz) and for very-high frequencies (9 to 16 kHz) for each ear. Pitch and loudness matches and pitch matching ability were assessed as within-subject factors by a mixed ANOVA between groups. When interactions involving groups were significant, Tukey post-hoc tests were conducted. The test-retest reliability between sessions was assessed using mixed ANOVAs with Group as the between-subject factor, and Frequency and Session (Test/Retest) as the within-subject factors. When interactions involving Session were significant, repeated-measure ANOVAs and paired sample t-tests were conducted. Pearson product-to-moment correlations were also used to assess within-trial reliability. Binary logistic regression was used to assess sensitivity and specificity of the psychoacoustic measures. The dependent variable was the presence of tinnitus irrespective of musicianship (Tinnitus/No tinnitus). Predictor variables were the two predominant pitches (the two highest likeness rating scores) and loudness match at these predominant frequencies. Statistical analyses were performed with SPSS 18.0 for Windows (Chicago, IL, USA).

## Results

### Tinnitus pitch matching using the likeness ratings

**Figure 3:**
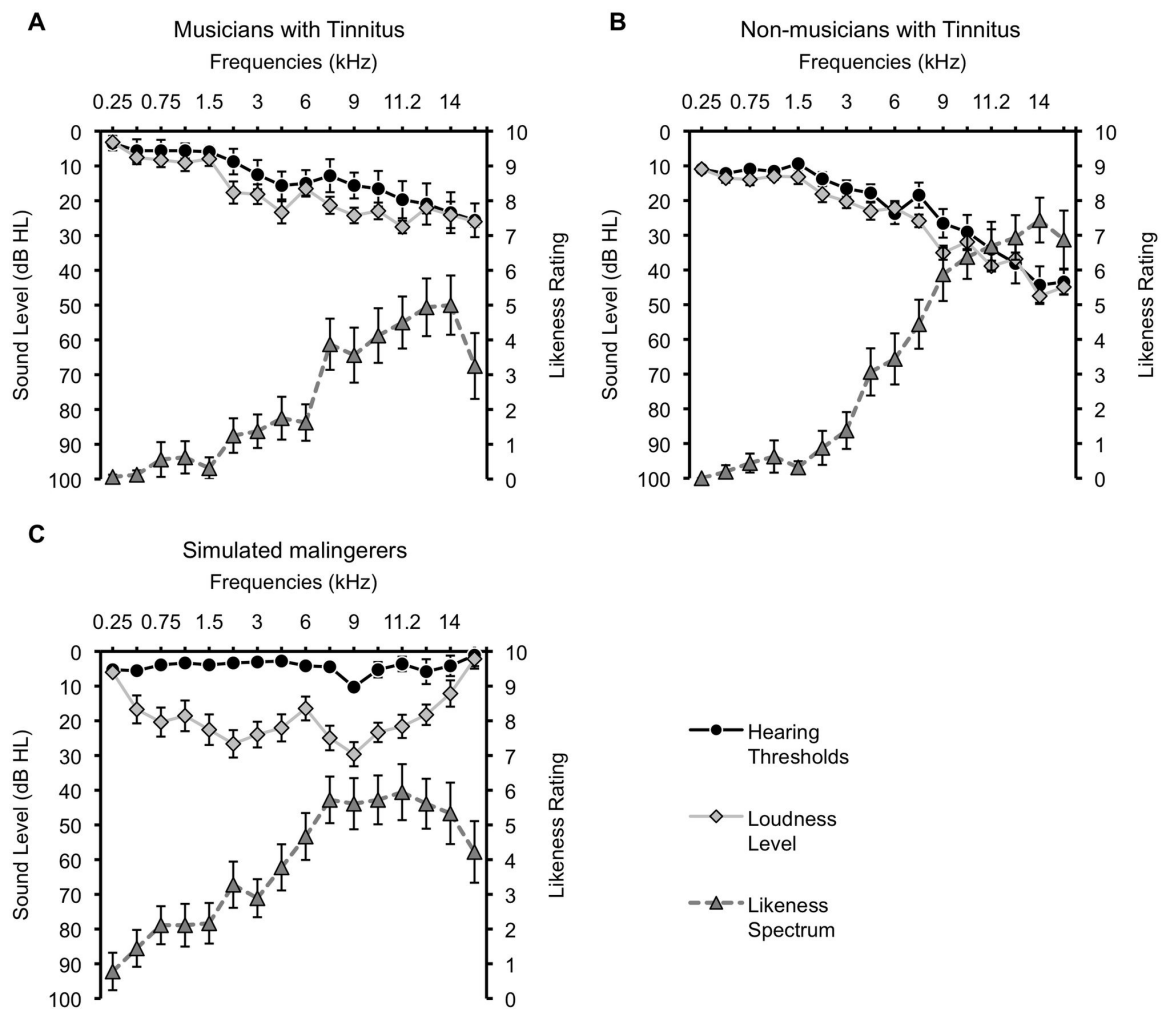
The tinnitus spectrum (gray dotted line) mirrors hearing loss for both musicians (A) and non-musicians (B). Pure-tone thresholds (black line) are reported for the right ear. All groups rated the predominant tinnitus pitch in the high frequencies (>8 kHz). For simulated malingerers (C), tinnitus loudness matching (clear line) is well above the one of tinnitus participants. Error bars represent the standard error of the mean.

**Figure 4:**
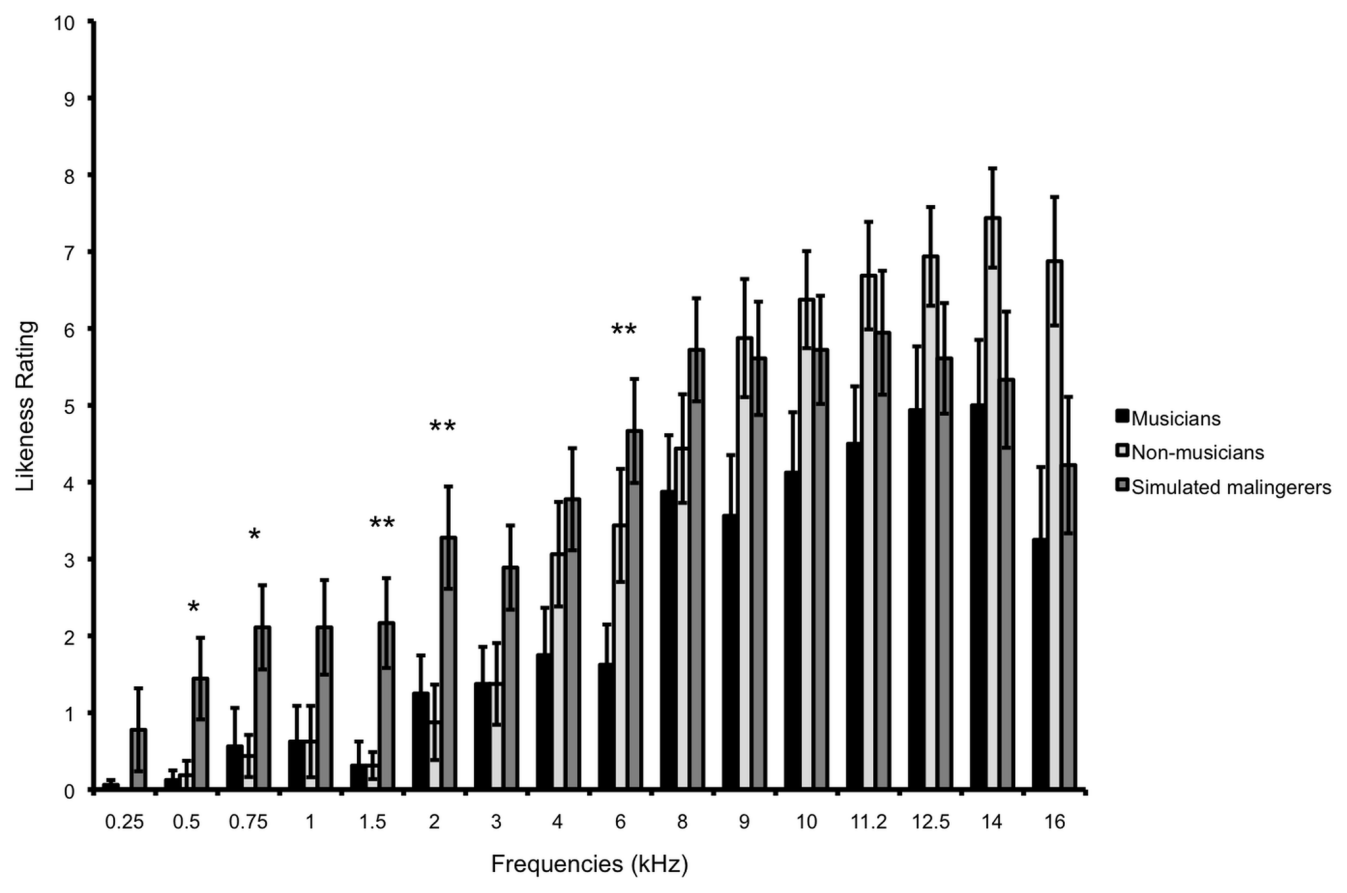
Likeness ratings for the three groups. Simulated malingerers differed from tinnitus participants in the low frequency range (*p<.05; **p<.01). Error bars represent the standard error of the mean.

A significant two-way interaction between Frequency and Group was found [*F*(30,705)=2.07; *p*=.001]. Musicians and non-musicians rated the pitch of their tinnitus very similarly, differing only for 16 kHz (means of 3 and 7, respectively, *p*=.019), whereas simulated malingerers rated lower frequencies as being more like their tinnitus (see Figures 3 and 4) than did musicians (at 0.5 kHz, 1.5 kHz, 2 kHz, and 6 kHz, all *p* values between .029 and .005) and non-musicians (at 0.5 kHz, 0.75 kHz, 1.5 kHz, and 2 kHz, all *p* values between .039 and .007). At retest, only the main effect of Frequency [*F*(15,375)=50.78; *p*<.001] was significant. 

For likeness rating reliability, the three-way interaction between Session (Test vs. Retest), Frequency, and Group was marginally significant [*F*(30,375)=1.49; *p*=.05]. This was due to a significant difference between the two sessions at 11.2 kHz for musicians (*p*=.047 by paired sample t-tests) (see Table 2). 

**Table 2:** Tinnitus likeness ratings and loudness matching differences (standard deviation) between test and retest for the three groups.

	**Mean likeness rating difference in numerical rating value**			**Mean loudness matching difference in dB SL**		
**Frequency in kHz**	**Musicians**	**Non-musicians**	**Simulated malingerers**	**Musicians**	**Non-musicians**	**Simulated malingerers**	
**0.25**	0 (0)	0 (1)	1 (1)	0 (1)	1 (2)	0 (13)	
**0.5**	0 (0)	1 (1)	1 (2)	0 (1)	2 (3)	6 (18)	
**0.75**	0 (1)	1 (2)	1 (3)	1 (5)	1 (4)	4 (21)	
**1**	0 (1)	1 (2)	1 (2)	1 (8)	3 (6)	5 (28)	
**1.5**	0 (0)	1 (3)	2 (2)*	4 (8)	5 (8)	3 (29)	
**2**	0 (2)	1 (2)	0 (4)	1 (13)	5 (7)	7 (34)	
**3**	0 (2)	0 (3)	1 (3)	1 (7)	1 (4)	6 (29)	
**4**	1 (3)	0 (2)	2 (3)	2 (2)*	2 (4)	11 (20)	
**6**	1 (3)	1 (2)	0 (3)	1 (5)	3 (4)	18 (12)**	
**8**	1 (3)	2 (3)	2 (3)	0 (9)	2 (8)	16 (18)*	
**9**	3 (5)	0 (3)	0 (2)	1 (14)	2 (7)	14 (14)*	
**10**	2 (4)	0 (3)	0 (2)	2 (6)	1 (7)	21 (16)**	
**11.2**	2 (3)*	1 (2)*	1 (2)	3 (12)	1 (5)	18 (16)**	
**12.5**	1 (4)	1 (1)	0 (2)	2 (6)	1 (9)	19 (14)**	
**14**	1 (3)	1 (2)	1 (2)	5 (11)	2 (8)	22 (15)**	
**16**	4 (6)	0 (4)	1 (3)	3 (8)	1 (7)	32 (13)***	

The asterisks represent *p*-values of *p<.05, **p<.01, ***p<.001, for paired-sample t-tests between test and retest for each group. Paired sample t-tests were used as post-hoc tests following up a three-way interaction between Groups X Session X Frequency (Mixed ANOVA). The interaction was driven mainly by the simulated malingerers group, who were not consistent between test and retest at high frequencies (>6 kHz). The dotted line represents the border between standard audiometric measurements (<8 kHz) and very-high frequencies (>8 kHz).

### Tinnitus loudness matching using the likeness ratings

A significant two-way interaction between Frequency and Group was also found [*F*(30,705)=2.16; *p*<.001]. Simulated malingerers rated the loudness of their tinnitus much higher than did both musicians and non-musicians at all frequencies except 0.25 kHz (all *p* values between .004 and <.001 by post-hoc tests) (see Figure 3). From 0.25 to 16 kHz, the mean tinnitus loudness was 4 ± 2 dB SL for musicians, 3 ± 2 dB SL for non-musicians, and 28 ± 2 dB SL for simulated malingerers. There was no significant difference between musicians and non-musicians. Retest data showed a main Group effect [*F*(2,25)=9.36; *p*=.001]. Again, simulated malingerers rated loudness much higher than did the musicians (*p*=.001) and non-musicians (*p*=.008) (mean = 18 ± 3 dB SL). Musicians and non-musicians did not differ in their loudness matches, with means of 1 ± 3 dB SL and 4 ± 3 dB SL, respectively, from 0.25 to 16 kHz. 

For loudness matching reliability, an interesting result emerged. The three-way interaction between Session, Frequency, and Group was significant [*F*(30,375)=1.99; *p*=.002]. Two-way ANOVAs were conducted separately for the three groups. For simulated malingerers, a significant interaction between Session and Frequency was found [*F*(15,135)=3,49; *p*<.001]. Simulated malingerers’ loudness ratings differed from test to retest at all frequencies from 6 to 16 kHz (*p*<.05 for paired-sample t-tests) (see Table 2). This was not the case for musicians and non-musicians, whose mean loudness test-retest ratings were stable at all frequencies except for musicians at frequency 4 kHz (hence the three-way interaction).

### Predominant tinnitus pitch and loudness with the likeness ratings

Table 3 summarizes the results of the predominant tinnitus pitch and its loudness for the three groups at test (Table 3A) and retest (Table 3B). Tinnitus predominant pitch differed between groups (*p*=.002) only at the first test session. Non-musicians rated their predominant tinnitus pitch slightly higher than did musicians (means = 14.2 kHz and 10.3 kHz, respectively, *p*=.027) and simulated malingerers (mean = 8.9 kHz, *p*=.001), but simulated malingerers and musicians did not differ (*p*=.58). Loudness at the predominant tinnitus pitch differed between groups for both test sessions (*p*<.001). Simulated malingerers rated the tinnitus loudness much higher than did musicians and non-musicians at both test (mean differences = 27.5 and 37.5 dB SL, respectively, both *p*<.001 by post-hoc comparisons) and retest (mean differences = 23.7 and 20.5 dB SL, respectively, *p*<.001 and *p*=.003). 

**Table 3:** Psychoacoustic characteristics of tinnitus pitch and loudness (standard deviation) for musicians with tinnitus, non-musicians with tinnitus, and simulated malingerers, at test (A) and retest (B), assessed by the likeness rating method.

**A**	**Musicians**	**Non-musicians**	**Simulated malingerers**	***p*-Value**
Mean predominant tinnitus pitch in kHz	10.3 (4.6)	14.2 (1.7)	8.9 (5.0)	.002
Mean loudness at the predominant pitch in dB SL	11.3 (12.6)	1.2 (5.9)	38.8 (18.9)	<.001
**B**	**Musicians**	**Non-musicians**	**Simulated malingerers**	***p*-Value**
Mean predominant tinnitus pitch in kHz	12.9 (3.9)	13.9 (1.7)	9.1 (3.8)	n.s.
Mean loudness at the predominant pitch in dB SL	-1.1 (7.5)	2.0 (6.7)	22.5 (17.8)	<.001

### Loudness, not pitch, predicts tinnitus malingering

When loudness at the two predominant tinnitus pitches was used as a predictor of the presence or absence of tinnitus, the model was very successful (*R* ^2^ = .752, overall percentage = 94.0%), correctly identifying 93.8% of tinnitus participants (i.e., sensitivity, n=30), while correctly rejecting 94.4% of participants without tinnitus (i.e., specificity, n=17). The model was much less successful (*R* ^2^ = .163, overall percentage = 70.0%) when using the two predominant tinnitus pitches, correctly identifying 90.6% of tinnitus participants (i.e., sensitivity, n=29) while correctly rejecting only 33.3% of participants without tinnitus (i.e., specificity, n=6). At retest, better predictive values were again found for tinnitus loudness than pitches (*R* ^2^ = .693 vs. .243, overall percentages of 84.6% vs. 73.1%), with sensitivity of 93.8% (vs. 87.5%) and specificity of 70.0% (vs. 50.0%). 

### Tinnitus matching using the slider method

A mixed ANOVA between tinnitus matching trials (3) and Group was performed, and a main Group effect was found [*F*(2,47)= 6.40; *p*=.003]. Results were similar to those obtained with the likeness ratings at both test and retest: at test non-musicians described a mean pitch higher than musicians (with means of 13.5 ± 3.0 kHz and 9.6 ± 5.2 kHz, respectively, *p*=.049 by post-hoc comparisons) and than simulated malingerers (mean = 8.0 ± 5.2 kHz, *p*=.003), but there was no significant difference between musicians and simulated malingerers (see table 4A). At retest, no significant main effects or interactions were found (see table 4B). For pitch matching reliability, there was no interaction between sessions; however, a main effect of Group was significant [*F*(2,25)=3.95; *p*=.032]. For both sessions, non-musicians had a mean tinnitus pitch higher than did simulated malingerers (with means of 13.9 ± 1.5 kHz and 10.0 ± 4.6 kHz, respectively, *p*=.025, by post-hoc comparisons). Table 5 shows the inter-trials reliability between the groups at test (Table 5A) and retest (Table 5B). All groups were consistent in their responses of tinnitus pitch in both sessions (all *rs* > .80, *p*<.01).

**Table 4:** Pitch matching values in kHz (standard deviation) for the three groups, at test (A) and retest (B) for both methods.

**Tinnitus pitch matching**	**Musicians**		**Non-musicians**		**Simulated malingerers**	
**A**	**Mean in kHz**	***p*-Value**	**Mean in kHz**	***p*-Value**	**Mean in kHz**	***p*-Value**
Likeness rating method	10.3 (4.6)		14.2 (1.7)		8.9 (5.0)	
Slider method	9.6 (5.2)		13.5 (3.0)		8.0 (5.2)	
Difference between methods	0.8 (3.6)	.40	0.7 (3.2)	.40	1.0 (3.9)	.31
**B**	**Mean in kHz**	***p*-Value**	**Mean in kHz**	***p*-Value**	**Mean in kHz**	***p*-Value**
Likeness rating method	12.8 (3.9)		13.9 (1.7)		10.1 (3.8)	
Slider method	11.9 (3.9)		13.9 (1.5)		10.0 (4.6)	
Difference between methods	1.0 (4.8)	.56	.02 (1.3)	.96	.11 (4.4)	.94

**Table 5:** Pearson product-to-moment correlations of the inter-trial reliability of tinnitus pitch matching using the slider for the three groups, at test (A) and retest (B).

	**Musicians**			**Non-musicians**			**Simulated malingerers**		
**A**	**Mean difference in kHz (SD)**	***r***	***p***	**Mean difference in kHz (SD)**	***r***	***p***	**Mean difference in kHz (SD)**	***r***	***p***
Trial 1 – Trial 2	0.5 (1.4)	.96	<.001	0.4 (1.6)	.89	<.001	0.4 (2.1)	.92	<.001
Trial 2 – Trial 3	0.2 (1.1)	.98	<.001	0.5 (1.7)	.92	<.001	0.4 (1.3)	.97	<.001
Trial 1 – Trial 3	0.3 (1.4)	.97	<.001	0.1 (0.9)	.97	<.001	0.04 (2.0)	.92	<.001
**B**	**Mean difference in kHz (SD)**	***r***	***p***	**Mean difference in kHz (SD)**	***r***	***p***	**Mean difference in kHz (SD)**	***r***	***p***
Trial 1 – Trial 2	0.4 (2.0)	.90	.001	0.2 (0.6)	.94	<.001	0.8 (1.4)	.98	<.001
Trial 2 – Trial 3	0.3 (2.4)	.81	.008	0.08 (0.8)	.89	.001	0.2 (1.1)	.97	<.001
Trial 1 – Trial 3	0.7 (2.6)	.80	.009	0.3 (0.8)	.91	.001	0.6 (1.1)	.99	<.001

### Predominant tinnitus pitch does not differ from one method to the other

The predominant tinnitus pitch was compared between the two matching methods using paired sample t-tests. Results are displayed in Table 5 for each group for both sessions. The mean tinnitus pitch differences were not significant for musicians, non-musicians, and simulated malingerers at test (Table 4A) or at retest (Table 4B). 

### No difference between tonal and noise tinnitus types

The psychoacoustic characteristics (predominant pitch, loudness) and the psychological aspects (VAS and THQ) of tinnitus were compared between tinnitus reported as “tonal” and “noise” types using paired sample t-tests and ANOVA. Simulated malingerers were not included in this analysis. Results are shown in Table 6. Pitch and loudness at the predominant pitch and the distress measured through VAS and THQ scores did not differ between the two subgroups. The number of predominant frequencies tended to be higher by 1 in noise tinnitus than in tonal tinnitus, but the difference did not reach significance.

**Table 6:** Psychoacoustic characteristics of tinnitus (pitch, loudness) and psychological distress (VAS, THQ) (standard deviation) between reported tonal tinnitus and noise tinnitus.

**Reported type of tinnitus**	**Tonal**	**Noise**	***p*-Value**
N	24	8	
Number of predominant frequencies	1.4 (0.6)	2.9 (1.9)	.07
Predominant pitch in kHz (likeness)	12.8 (3.9)	10.7 (3.8)	n.s.
Predominant pitch in kHz (slider)	11.8 (4.6)	10.3 (4.6)	n.s.
Loudness at the predominant pitch in dB SL	6.6 (12.4)	5.4 (4.9)	n.s.
VAS score	37.7 (5.5)	31.1 (9.0)	n.s.
THQ score	26.6 (16.5)	23.8 (20.1)	n.s.

### Tinnitus percept does not correlate with VAS or THQ

Table 7 shows the correlations among all VAS, the THQ, and psychoacoustic predominant pitch and loudness at the predominant pitch for tinnitus participants. Simulated malingerers were not included in this analysis. All VAS, including loudness, were highly correlated with THQ scores, but not with psychoacoustic pitch and loudness. 

**Table 7:** Correlations among visual analog scales scores, Tinnitus Handicap Questionnaire scores, and psychoacoustic pitch and loudness.

**Visual analog scales**	**THQ total score**		**Predominant pitch**		**Loudness at the predominant pitch**	
	*r*	*p*-Value	*r*	*p*-Value	*r*	*p*-Value
Question 1						
How annoying is your tinnitus **usually**?	.75**	<.001	.25	.22	-.09	.68
Question 2						
How annoying is your tinnitus **now**?	.70**	<.001	.31	.13	-.08	.70
Question 3						
How loud is your tinnitus **usually**?	.67**	<.001	.30	.13	-.17	.40
Question 4						
How loud is your tinnitus **now**?	.60*	=.001	.29	.16	-.10	.62
Question 5						
How much attention do you spend on your tinnitus **usually**?	.72**	<.001	.32	.11	-.24	.23

The asterisks represent *p*-values of *p<.01, **p<.001.

### Ability to match pitch to external tones

**Figure 5:**
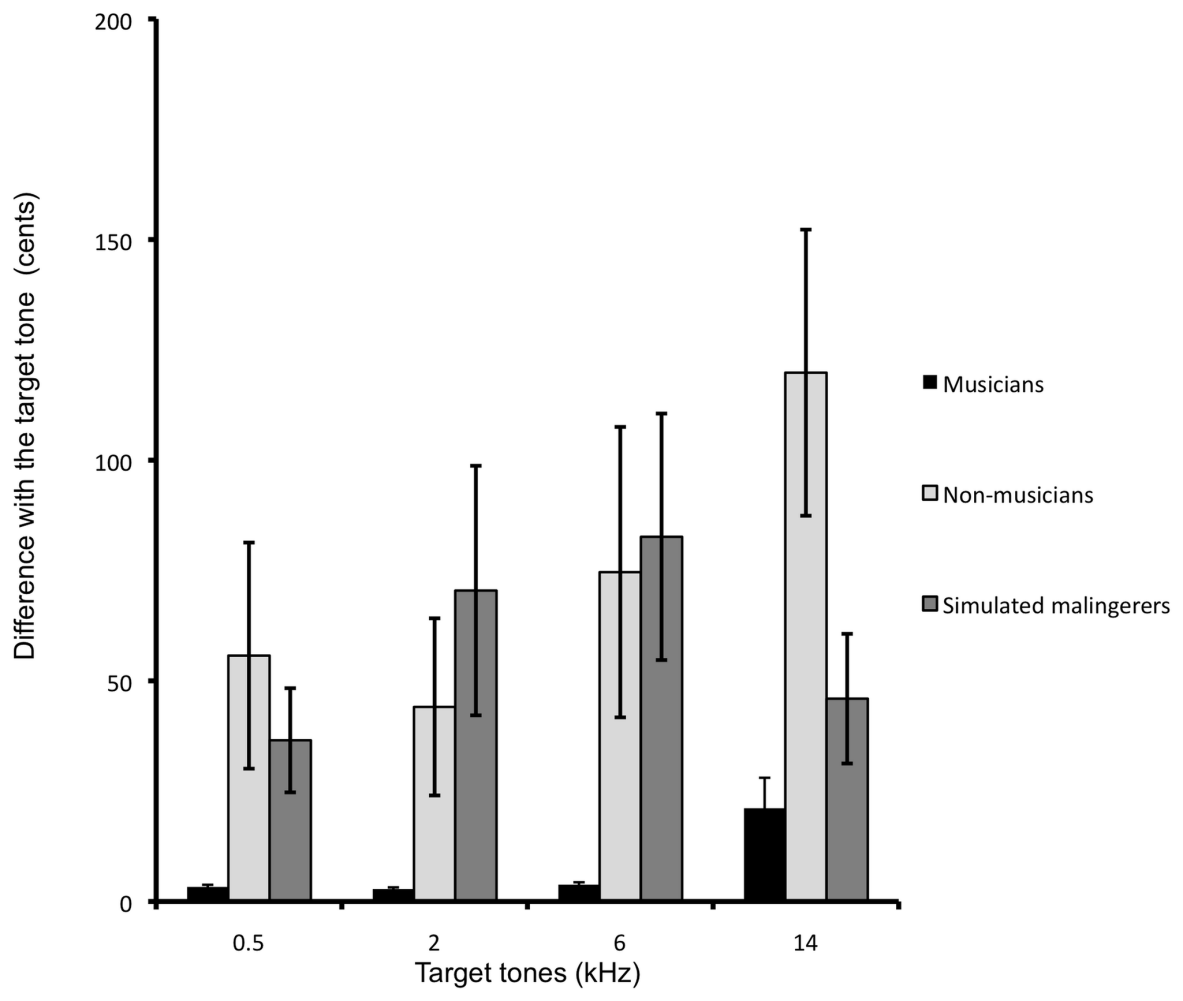
Differences in cents between target and matched frequencies using the slider for musicians, non-musicians, and simulated malingerers (test session). Error bars represent the standard error of the mean.

Figure 5 shows differences between target and matched frequencies using the slider for musicians, non-musicians, and simulated malingerers. Since variability among musicians’ responses was as much as 74 times smaller than within the two other groups and Levene’s test for homogeneity of variances was significant for all target tones (all ps<.001), non-parametric Kruskal-Wallis and Mann-Whitney U tests were used to test group differences for each target tones. Differences among groups were significant for all four target tones (all *p*s between <.001 and .015). Musicians were better able to match target tones than were non-musicians at all frequencies (all *p*s between <.001 and .008) and than simulated malingerers at all frequencies except 14 kHz (*p*s between .002 and .004, *p*=.13 for 14 kHz). Non-musicians and simulated malingerers did not differ from one another at any frequency (all *p*s between .07 and .72). Overall, the mean difference in cents was 7.8 (SE: 17.5) for musicians compared to 76 (SE: 18.1) for non-musicians —almost ten times greater than musicians —and 59 (SE: 16.5) for simulated malingerers. At retest, differences among groups were significant at all target tones except 14 kHz (all *p*s between .003 and .05; *p*= .57 at 14 kHz). Musicians differed from non-musicians and simulated malingerers at all frequencies except 14 kHz (all *p*s between .002 and .05; ps= .69 and .23 at 14 kHz, respectively). Again, non-musicians and simulated malingerers did not differ from one another at any frequency (all *p*s between .12 and .69). At retest, the mean difference in cents was 6.7 (SE: 16.5) for musicians, compared to 51.9 (SE: 16.5) for non-musicians and 36.2 (SE: 16.5) for simulated malingerers.

## Discussion

Herein, we reported several novel findings in support of the psychoacoustic assessment of tinnitus for tinnitus diagnosis and characterization.

### Psychoacoustic assessment improves tinnitus diagnosis

Using a participant-directed likeness rating method to match tinnitus pitch and loudness over a wide frequency spectrum (from 0.25 to 16 kHz), we found that musicians and non-musicians rated the pitch of their tinnitus very similarly, with low likeness ratings in the low frequencies rising slowly toward the highs. Likewise, musicians and non-musicians rated the SL loudness of their tinnitus no differently over the whole frequency span and in the SL range usually described (< 5 dB SL). In sharp contrast, even relying on their past—though fleeting—experience of tinnitus, simulated malingerers rated their tinnitus as being composed of lower pitches and at a much higher loudness than did tinnitus participants over the entire range of frequencies except 0.25 kHz. Our findings support and extend previous findings showing lower pitch matches [39,40] and higher loudness in SL [42] in simulated malingerers but contradict those reporting lower loudness or no difference between tinnitus and no-tinnitus participants [39,40,43]. The robustness of our data was further corroborated, however, by a retest session that took place six months on average after the first session, compared to less than a month in previous studies [39,40,42]. While reliability of pitch matching was similar in our three groups, loudness reliability was excellent among musicians and non-musicians but much less reliable among simulated malingerers. It is striking that the last group’s loudness ratings were different from test to retest over a broad range of frequencies, especially those in the very high frequencies, which are not routinely assessed during audiological testing. This finding highlights the importance of assessing frequencies above 8 kHz for tinnitus diagnosis and puts forth the potential value of loudness as a parameter to distinguish individuals simulating tinnitus from those who genuinely have tinnitus. 

### Tinnitus pitch identification

One important question addressed here was whether the likeness rating method could allow the extraction of one predominant tinnitus pitch that provides an advantage over no constraint on pitch selection. Predominant tinnitus pitch using the likeness ratings was higher for non-musicians than for both musicians and simulated malingerers. This is unsurprising since non-musicians displayed slightly higher very-high frequency thresholds and tinnitus pitch is usually in the frequency-band region of hearing loss [26,28,31,34]. Use of the slider yielded essentially the same results: non-musicians rated their tinnitus pitch as higher than both musicians and simulated malingerers, with no difference between the latter two. Strikingly, the comparison between these two extremely different methods indeed yielded no significant tinnitus pitch differences for any of the groups, therefore supporting the strength of the likeness rating method in extracting a predominant tinnitus pitch. Similar to what has been described in previous studies [31,33], it is remarkable that participants who reported noise tinnitus were able to identify a predominant pitch. Given that tonal tinnitus comprises a bandwidth that can be wider than noise tinnitus [31,33], the relevance of distinguishing tonal from noise tinnitus becomes questionable. More detailed differences among tinnitus spectra, such as type of hearing loss, may be more relevant to distinguishing subgroups of tinnitus patients [29].

Our data emphasize the appropriateness of the likeness rating method for assessing the tinnitus pitch of participants notwithstanding their musical backgrounds. Our study is the first to assess whether musical training could improve the assessment of tinnitus pitch. Indeed, although musicians were able to match external sine waves within a few cents, all three groups were consistent at matching their tinnitus pitch. 

### Tinnitus predictors

One of the most novel findings of this study is that when predominant pitch and loudness are extracted from the likeness ratings and compared as predictors for the presence of tinnitus, it is loudness, not pitch, that has the greatest predictive value. Psychoacoustic loudness at the predominant pitch is therefore a sensitive and specific measure of the tinnitus percept. This was shown here repeatedly by the fact that simulated malingerers rated higher loudness levels at many frequencies, and evinced loudness (not pitch) unreliability from test to retest, especially in the very-high frequency range. These results contradict a previous study [43], which found that lower loudness matches sensation level as sensitive factor of tinnitus absence. If loudness is rated greater and less reliably from one session to the next, clinicians may have an indication that tinnitus is not present. Therefore, implementing a likeness rating method similar to the touchscreen in clinical practice could potentially be a tool for discriminating tinnitus sufferers from malingerers. In 30 minutes of testing, the experimenter is able to measure both tinnitus pitch and tinnitus loudness and to report whether loudness matching is consistent with real tinnitus or simulation. If there is doubt, a retest would confirm greater loudness matches. Finally, unreliability between test and retest would provide a third opportunity to detect simulated tinnitus, especially at very-high frequencies. 

### Perspective: Tinnitus percept versus distress

Predictably, widely used visual analog scales or handicap questionnaires were not correlated to psychoacoustic parameters of tinnitus, a finding consistent with previous studies that found that visual analog scales are correlated with mood and distress rather than actual loudness in SL [50]. We therefore propose that psychoacoustic loudness should constitute an essential and complementary measure of tinnitus. In this regard, the literature on pain, to which tinnitus is often compared, is enlightening. The largely independent encoding, modulation [51], and brain networks for [52] sensory (pain intensity) and affective (pain unpleasantness) dimensions of pain suggest that it is a multidimensional response system that differentially encodes both qualities. Furthermore, psychological interventions involving emotions (such as cognitive behavioural therapy) modulate perceived pain unpleasantness more than perceived intensity of pain [53], whereas therapies involving distraction seem to modulate more directly perceived intensity of pain and not mood [54,55]. If we transfer this analogy to tinnitus, this means that sensory (percept) and affective (distress) dimensions of tinnitus would be separable. The lack of correlations between psychoacoustic loudness and distress shown in our study (and previous ones [56,57]) and evidence showing separable brain networks of tinnitus psychoacoustic loudness and distress, although still scarce [58], are both consistent with this idea. The implication is that interventions modulating mood, such as cognitive behavioural therapy, would act on the unpleasantness of tinnitus, which seems to be the case [59,60], whereas therapies that modulate attention, such as noise generators or neuromodulators of attention (see 61 for a discussion of the role of attention in tinnitus), would act mainly on tinnitus percept. One study has shown that alprazolam, a benzodiazepine that binds to GABA_A_ receptors, significantly reduced both tinnitus psychoacoustic loudness (3.6 dB on average) and loudness on a 10-point visual analog scale (1.5 points on average) after 12 weeks [62]. The systematic assessment of both tinnitus percept and distress would make the field progress by identifying which therapies act on distress only, both distress and percept, or percept only. The answer to this question has important implications about the underlying mechanisms of tinnitus and those involved in treatment efficacy. 
